# Molecular spectrum of laterally coupled quantum rings under intense terahertz radiation

**DOI:** 10.1038/s41598-017-10877-y

**Published:** 2017-09-05

**Authors:** Henrikh M. Baghramyan, Manuk G. Barseghyan, David Laroze

**Affiliations:** 1 Instituto de Alta Investigación, CEDENNA, Universidad de Tarapacá, Casilla 7D, Arica, Chile; 2 Department of Solid State Physics, Yerevan State University, Alex Manoogian 1, 0025 Yerevan, Armenia; 3 Yachay Tech University, School of Physical Sciences and Nanotechnology, 00119 Urcuquí, Ecuador

## Abstract

We study the influence of intense THz laser radiation and electric field on molecular states of laterally coupled quantum rings. Laser radiation shows the capability to dissociate quantum ring molecule and add 2-fold degeneracy to the molecular states at the fixed value of the overlapping size between rings. It is shown that coupled to decoupled molecular states phase transition points form almost a straight line with a slope equal to two. In addition, the electric field direction dependent energy spectrum shows unexpected oscillations, demonstrating strong coupling between molecular states. Besides, intraband absorption is considered, showing both blue and redshifts in its spectrum. The obtained results can be useful for the controlling of degeneracy of the discrete energy spectrum of nanoscale structures and in the tunneling effects therein.

## Introduction

Quantum rings (QR)^1^ exhibit a unique density of states^2^ for charge carriers and hence a vast variety of physical properties, which are cardinally different from those of singly-connected structures like quantum dots (QDs). QRs can be grown by the various techniques: InAs/GaAs and InAs/InP QR have been obtained by the partial overgrowth (that is based on a partial capping and short time annealing process of self-assembled InAs QDs) during molecular beam epitaxy (MBE)^3, 4^. MBE droplet epitaxy has been used for the formation of GaAs/AlGaAs QRs^5^, and metal-organic chemical vapor deposition for the growth of InAsSb/InP QRs^6^. For application purposes, especially interesting are the coupled assemblies of QRs^7, 8^, in which, due to the small stray field and high tunneling magnetoresistance ratio, nanoring-magnetic tunnel junctions offer superior prospects for very high density magnetic random access memory, recording medium, and other spintronic devices. The model calculations of wave function engineering in quantum dot-ring nanostructures (DRN)^9, 10^ have shown potential use in tunable or switchable microwave waveguides built with the help of arrays of DRNs with variable optical properties or in a single electron transistor based on a single DRN coupled to source and drain leads. Recently, an interesting model of polygonal few electrons QRs^11^ has been shown that Coulomb repulsion allows the formation of corner-localized states of electron pairs (or clusters) shifted to energies that were forbidden for non-interacting electrons. Besides, metamaterials made up of QRs have demonstrated negative refractive index while studying scattering data at microwave frequencies^12, 13^. The coupling in vertically stacked layers of QRs has also demonstrated potential to enhance single-mode operation of laser diodes^14^ and influenced the carrier transfer in both vertical and lateral directions^15^. In the light of the mentioned above works one can consider coupled arrangements of semiconductor QRs as a model for artificial molecules similar, for example, to the chain of molecules composed by benzene rings^16, 17^: indeed, in the ground states for laterally coupled QR molecules of rings of different radii the magnetic field transfers the electron charge from one ring to the other^18^. Also, the inter-ring coupling is responsible for the reduction of persistent currents and magnetic dipole moments in a two-dimensional array of multiple QRs^19^ and, as proposed in ref. 20, even in the absence of the net flux Aharonov-Bohm oscillations can be obtained in QR molecules. In addition, calculations of persistent currents in a QRs modeled by the Holstein-Hubbard-Rashba^21^ have demonstrated that the electron-phonon interactions reduce the persistent current, while the Rashba coupling enhances it and the temperature smoothens the persistent current curve. Besides, it has shown that the indium’s concentration is fundamental to determine the confined electron wave functions in a set of QDs connected with QRs^22^. Therein, for the calculated ground and first excited states the wave functions were confined within a single dot without an evidence of coupling between QDs^22^.

On the other hand, it is quite attracting to study inter-ring coupling under the excitation of intense terahertz (THz) radiation, in contrast to the solid-state lasers used, for example, in refs 14 and 15. Intense THz radiation created by high-power lasers, such as optically pumped THz molecular or free electron lasers, gives rise to a variety of nonlinear phenomena whose characteristic features are basically different from the corresponding effects at microwave frequencies as well as in the range of visible radiation^23^. It is because in the electron-radiation interaction the transition from semiclassical physics with a classical field amplitude to the fully quantized limit with photons occurs at THz frequencies. In fact, in ref. 24. a photon-mediated sequential tunneling process has been observed in a ${\mathrm{GaAs}/\mathrm{Al}}_{0.3}{{\rm{Ga}}}_{0.7}{\rm{As}}$ superlattice, that is a consequence of a new conduction channel opened by the THz electric field, in which an electron can tunnel from the ground state in one quantum well (QW) to an excited state in the neighboring well with the absorption of a photon. In addition, under the illumination of intense monochromatic THz radiation the photon-assisted electron tunneling has been studied in single-C_60_-molecule transistors^25^, indicating that when the incident THz intensity is a few tens of mW, the THz field induced at the molecule exceeds 100 kV/cm, which is enhanced by a factor of ~10^5^ from the field in the free space. Recently, observations of high-order-sideband generation in QWs resulting from electron-hole recollisions have been reported^26^, which suggest a new mechanism for the ultrafast modulation of light. It is also worth to mention the photoresponse of AlGaN/GaN high electron mobility transistors to the THz radiation of low $\mathrm{(15}\,{\mathrm{mW}/\mathrm{cm}}_{2})$ and high (${\rm{up}}\,{\rm{to}}40\,{\mathrm{mW}/\mathrm{cm}}_{2}$) intensities^27^ and the observation of a THz radiation-induced photon drag effect in n and p-type ${{(\mathrm{Bi}}_{1-{\rm{x}}}{{\rm{Sb}}}_{{\rm{x}}})}_{2}{{\rm{Te}}}_{3}$ three-dimensional topological insulators^28^.

The laser-driven electronic states has been theoretically studied in various semiconductor nanostructures, resulting in many interesting effects: in QWs and quantum wires laser field has lead to the change of the density of states’ profile to a profile typical to quantum wires^29^ and QDs correspondingly^30, 31^, in-plane THz field in QWs has induced THz replicas of the (dark) 2p exciton and THz sidebands of the 1 s exciton^32^, a model has been proposed to reform a single QW to a double QW by the appropriate selection of the laser field frequencies as well as the intensities^33^. Moreover, strong laser field assisted transport properties in semiconductor nanostructures have been carried out^34–36^. In this framework, it has been shown that dressing field leads to a giant increase in the conductivity of two-dimensional electron gas in GaAs QWs^35^. In ref. 36. the authors have developed the theory of spin-dependent transport through a Datta-and-Das spin transistor in the presence of a high-frequency laser field. They have demonstrated that the laser field can lead to the renormalization of spin-orbit coupling constants that vary the conductivity of the spin transistor. Furthermore, in graphene it has shown that the stationary electronic transport strongly depends on parameters of the dressing field^37^. In fact, a circularly polarized field monotonically decreases the isotropic conductivity of graphene, whereas a linearly polarized one increases both the giant anisotropy of conductivity as well as the oscillating behavior of the conductivity when field intensity is increased.

Respect to the theoretical studies on laser-driven energy states in quantum rings have been carried out, to the best of our knowledge, only in single ones^38–40^. In particular, it has been shown that the intraband absorption spectrum can be shifted depending on the incident light polarization direction^38^, and that the inclusion of a constant electric field can lower the absorption strength more than two times^39^. In addition, Aharonov-Bohm effect has been observed for excitons in a single semiconductor QR dressed by a circularly polarized light, showing a physical nonequivalence of clockwise and counterclockwise exciton rotations in the ring^40^. The aim of the present work is to study the one-electron molecular system of laterally aligned ${\text{GaAs}/\text{Ga}}_{0.7}{{\rm{Al}}}_{0.3}{\rm{As}}$ QRs in the high-frequency approximation of Floquet method. In particular, we show that the influence of intense THz laser field can dissociate (decouple) the molecule, while the material overlapping size of the rings can be kept fixed. In addition, the energy spectrum of the molecular system demonstrates a new level of degeneracy under the influence of intense THz laser field. We have also considered the lateral electric field and obtained that it opens new channels for the transitions in the intraband absorption. We believe, the mentioned properties of Floquet quasi-states in laterally aligned QRs in the presence of a lateral electric field, is new and aimed to partially fill the gap of QR’s physics.

The structure of the paper is as follow. First, we state a problem providing the theory for the intense THz laser field interaction with QR molecule, and then we discuss the results of laser field induced decoupling of the molecule and 2-fold degeneracy induced by laser field and electric field effect on that degeneracy. Afterward, the intraband absorption is discussed under the laser and electric fields impact and the results of the electric field direction changing are given. Finally, in conclusions, a synopsis of the main results is given, and a method of numerical calculations is presented.

## Problem

The one-electron motion is considered two-dimensional and confined only in the plane of the rings, supported by the big difference of lateral (radii) and cross-sectional (heights) sizes of the QRs: the latter can be 10 times smaller according to ref. 5. In such situation, the corresponding time-dependent Schrödinger equation describing the system is given by

1 $$[\frac{1}{2m}{({\widehat{{\bf{p}}}}_{\perp }-\frac{e}{c}{\bf{A}}({\bf{r}},t))}^{2}+V({{\bf{r}}}_{\perp })-e{\bf{F}}\cdot {{\bf{r}}}_{\perp }]{\rm{\Phi }}({{\bf{r}}}_{\perp },t)=i\hslash \frac{\partial }{\partial t}{\rm{\Phi }}({{\bf{r}}}_{\perp },t)\,,$$

where $m=0.067\,{m}_{0}$ is the GaAs effective mass^41^, *m*_0_ is the free-electron mass, ${\widehat{{\bf{p}}}}_{\perp }$ is the two-dimensional momentum operator in XOY plane, *e* is the charge of an electron, *c* is the speed of light, **A** is the laser field vector potential, **F** is the strength of the uniform electric field, and *ħ* is the Planck constant. Working in dipole approximation^33, 42^, the vector potential will be spatially independent ${\bf{A}}({\bf{r}},t)\approx {\bf{A}}(t)={A}_{0}\,\cos \,\mathrm{(2}\pi \nu t){\widehat{{\bf{e}}}}_{{\boldsymbol{x}}}$. Here simple harmonic dependence is considered for the laser field, with ${\widehat{{\bf{e}}}}_{x}$ unit vector of polarization and ${A}_{0}={E}_{0}/\mathrm{(2}\pi \nu )$ is defined by the field strength *E*_0_ and frequency *v*. It is also non-resonant with GaAs bandgap according to $h\nu  < {E}_{{\rm{gap}}}^{{\rm{GaAs}}}=367\,{\rm{THz}}$ condition^43^. $V({{\bf{r}}}_{\perp })$ confining potential is 0 in ${R}_{in} < \sqrt{{(x\pm d\mathrm{/2)}}^{2}+{y}^{2}} < {R}_{out}$ (here ≪−≫ is in *x* > 0 half plane and ≪+≫ in $x\le 0$) and $257\,{\rm{meV}}$ ^44^ elsewhere, such that *d* is the distance between the centers of QRs, and $({R}_{in},{R}_{out})$ are the fixed inner and the outer radius, respectively. Here, we assume that the radii are ${R}_{in}\mathrm{=10}\,{\rm{nm}}$ and ${R}_{out}=50\,{\rm{nm}}$ ^5^.

Having dipole approximation already considered, Kramers-Henneberger (KH)^45, 46^ unitary transformation can be applied:

2 $${\rm{\Psi }}({{\bf{r}}}_{\perp },t)=\exp [(\frac{i}{\hslash }){\boldsymbol{\alpha }}\cdot {\widehat{p}}_{\perp }]\times \exp [\frac{i}{\hslash }\frac{{e}^{2}}{2m{c}^{2}}\int {{\bf{A}}}^{2}(t^{\prime} )dt^{\prime} ]{\rm{\Phi }}({{\bf{r}}}_{\perp },t),$$

where

3 $${\boldsymbol{\alpha }}(t)=-\frac{e}{mc}\int {\bf{A}}(t^{\prime} )dt^{\prime} $$

is the displacement vector of an electron due to its quiver motion in the laser field^42^. Let us remark that KH transformation moves the time dependence from the vector potential **A** to the scalar potentials. It is equivalent to change from the laboratory frame of reference (related with the geometrical center of the laterally coupled QR structure) to the accelerated one, which follows the ***α***(*t*) quiver motion of the election:

4 $$[\frac{{\widehat{{\bf{p}}}}_{\perp }^{2}}{2m}+V({{\bf{r}}}_{\perp }+{\boldsymbol{\alpha }}(t))-e{\bf{F}}\cdot ({{\bf{r}}}_{\perp }+{\boldsymbol{\alpha }}(t))]\Psi ({{\bf{r}}}_{\perp },t)=i\hslash \frac{\partial }{\partial t}\Psi ({{\bf{r}}}_{\perp },t)\,\mathrm{.}$$

Further, for the quiver motion displacement $\alpha (t)={\alpha }_{0}\,\sin \,\mathrm{(2}\pi \nu t)$ is obtained. Hereinafter, the influence of laser field is considered only by ${\alpha }_{0}=-(e/(m{\varepsilon }_{h}^{\mathrm{1/4}}{\nu }^{2}))\sqrt{I/(2c{\pi }^{3})}$ parameter, that comprises both the intensity *I* and frequency *v* of laser field, that can be chosen in a broad range in units of ${\mathrm{kW}/\mathrm{cm}}^{2}$ and THz correspondingly^23^, and *ε*_*h*_ = 10.9 is the high-frequency dielectric constant in GaAs^41^.

Since Eq. (4) is a linear partial differential equation with periodic coefficients, it can be treated with the non-perturbative Floquet method^47^. This method was originally used for atoms under intense laser radiation^42^. Afterward, it has been extensively used for semiconductor low-dimensional structures^29–31, 33, 38, 39, 48^. In addition, Floquet states in the continuum have been calculated for a quantum particle on a one-dimensional tight-binding lattice driven by an AC field^49^. In general, in the framework Floquet theory one tries to find the solution of Eq. (4) in terms of an infinite Fourier series of wave function $\Psi ({r}_{\perp },t)$. In addition, the potential terms $V({{\bf{r}}}_{\perp }+{\boldsymbol{\alpha }}(t))$ and $-{e}{\bf{F}}\cdot ({{\bf{r}}}_{\perp }+{\boldsymbol{\alpha }}(t))$ are also expanding in Fourier series. Therefore, they can be written as:

5 $${\rm{\Psi }}({{\bf{r}}}_{\perp },t)=\exp (-i(E/\hslash )t)\sum _{n=-\infty }^{n=\infty }\exp (-2i\pi n\nu ){\varphi }_{n}({{\bf{r}}}_{\perp }),$$

and

6 $$V({{\bf{r}}}_{\perp }+{\boldsymbol{\alpha }}(t))-e{\bf{F}}\cdot ({{\bf{r}}}_{\perp }+{\boldsymbol{\alpha }}(t))=\sum _{n=-\infty }^{n=\infty }\exp (-2i\pi n\nu )[{V}_{n}({{\bf{r}}}_{\perp },{\alpha }_{0})+{V}_{n}^{F}({{\bf{r}}}_{\perp },{\bf{F}})],$$

where

7 $${V}_{n}({{\bf{r}}}_{\perp },{\alpha }_{0})+{V}_{n}^{F}({{\bf{r}}}_{\perp },{\bf{F}})=\frac{1}{T}{\int }_{0}^{T}\exp (2i\pi n\nu )[V({{\bf{r}}}_{\perp }+{\boldsymbol{\alpha }}(\tau ))-e{\bf{F}}\cdot ({{\bf{r}}}_{\perp }+{\boldsymbol{\alpha }}(\tau ))]d\tau ,$$

and *T* is the period of the laser field. Inserting expressions (5) and (6) into Eq. (4), and after straightforward calculations one can find the Schrödinger equation is reduced to infinite system of time-independent coupled equations:

8 $$[\frac{{\widehat{{\bf{p}}}}_{\perp }^{2}}{2m}+{V}_{0}({{\bf{r}}}_{\perp },{\alpha }_{0})+{V}_{0}^{F}({{\bf{r}}}_{\perp },{\alpha }_{0})-(E+2\pi \nu n)]{\varphi }_{n}({{\bf{r}}}_{\perp })=-\underset{m\ne n}{\overset{m=\infty }{\sum _{m=-\infty }}}[{V}_{n-m}({{\bf{r}}}_{\perp },{\alpha }_{0})+{V}_{n-m}^{F}({{\bf{r}}}_{\perp },{\bf{F}})]{\varphi }_{m}({{\bf{r}}}_{\perp }\mathrm{).}$$

Using an iteration procedure, one can deal with the pervious set of equations. The detailed theoretical approach of it can be found in ref. 42. Here, we are interested only in the stationary states of an electron, which appear once the high-frequency approximation of Floquet method is applied^31, 33, 38, 39^. Namely, we assume that the frequency *v* of laser field is considered to be very high so that the electron only *feels* the time-averaged of the laser-dressed confining potential. High values of $(\nu ,I)$ can always be manipulated, meanwhile remaining in the range where dipole approximation and non-resonant laser field requirements are fulfilled^33^. For instance, it can be set up to ${\mathrm{MW}/\mathrm{cm}}^{2}$ for ${\text{GaAs}/\text{Ga}}_{0.7}{{\rm{Al}}}_{0.3}{\rm{As}}$ heterostructures^50^. Thus, in high-frequency approximation^51^ only zeroth-order solution of Eq. (8) contributes, which leads to the following time-independent Schrödinger equation^42^:

9 $$[\frac{{\widehat{{\bf{p}}}}_{\perp }^{2}}{2m}+{V}_{d}({{\bf{r}}}_{\perp },{\alpha }_{0})-e{\bf{F}}\cdot {{\bf{r}}}_{\perp }]{{\rm{\Psi }}}_{d}({{\bf{r}}}_{\perp })={E}_{d}{\Psi }_{d}({{\bf{r}}}_{\perp }),$$

where ${{\rm{\Psi }}}_{d}({{\bf{r}}}_{\perp })={\varphi }_{0}({{\bf{r}}}_{\perp })$ and ${V}_{d}({{\bf{r}}}_{\perp },{\alpha }_{0})={V}_{0}({{\bf{r}}}_{\perp },{\alpha }_{0})$ are called dressed wave function and dressed potential, respectively. Considering that the laser field is polarized along the *x*-axis, the dressed potential is reduced to

10 $${V}_{d}({{\bf{r}}}_{\perp },{\alpha }_{0})=\frac{1}{T}{\int }_{0}^{T}V(x+\alpha (\tau ),y){\rm{d}}\tau \mathrm{.}$$

Notice that under high-frequency approximation (*n* = 0) the electric field potential ${V}_{0}^{F}({{\bf{r}}}_{\perp },{\alpha }_{0})$ turns back to the original one because ${\int }_{0}^{T}{\bf{F}}\cdot {\boldsymbol{\alpha }}(\tau ){\rm{d}}\tau =0$. We remark that ${{\rm{\Psi }}}_{d}({{\bf{r}}}_{\perp })$ is the zeroth-order eigenfunction that defines laser-dressed Floquet states of an electron and that ${E}_{d}$ defines the corresponding eigenvalues (Floquet quasi-energies). Therefore, now the problem (9) can be interpreted as the stationary problem of a single electron in a complex potential ${V}_{d}({{\bf{r}}}_{\perp },{\alpha }_{0})-e{\bf{F}}\cdot {{\bf{r}}}_{\perp }$. The aforementioned equation is numerically solved to obtain the eigenvalues and eigenfunctions of the system. The details of the numerical methods will be presented in the Methods Section.

## Results and Discussion

**Figure 1:**
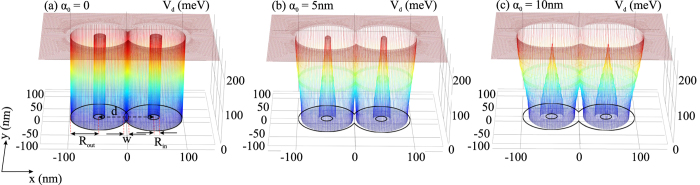
Laser-dressed confining potential for different values of *α*_0_ at *F* = 0. In the Supplementary materials the effect of the electric field is included in Figs S1 and S2.

In order to examine the main results of Eq. (9), let us first analyze the laser-dressed confining potential. Here, the potential is numerically calculated. Figure 1 shows *V*_*d*_ as a function of the space coordinates for different values of laser field parameter *α*_0_ at zero electric field. We can observe that the increment of *α*_0_ implies a bigger bleaching of the lower part of *V*_*d*_, which is a consequence of the contraction of the sizes of QRs. In fact, this contraction is greater in the laser field polarization direction. Notice that the contraction phenomenon has been analytically obtained in our previous works in the case of a single QR^38, 39^. The effect of the electric field on the potential can be found in the Supplementary Fig. S1. In the next subsections we will analyze the different effects of the parameters.

### Decoupling of QR molecule by laser field

**Figure 2:**
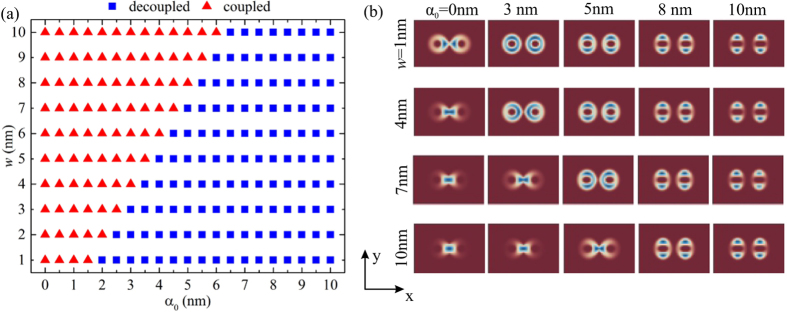
(**a**) *w* − *α*_0_ phase diagram of the coupled–decoupled transition and (**b**) probability density of the ground state for selected different values of *w* and *α*_0_.

One interesting consequence of the bleaching of the confining potential is the decoupling the QRs, or in other words, the dissociation of the molecule. The frame (a) of Fig. 2 shows $({\alpha }_{0},w)$ phase diagram that reflects the mentioned coupling-decoupling effect for the ground state, such that $w=2{R}_{out}-d$ is the overlapping region size of the QRs along the *x*-axis, as it is shown in Fig. 1. We numerically found that the transition from coupled to decoupled ring-localized states is almost linear with transition points forming a line with a slope *k* = 2. Selected images for probability densities are shown in the frame (b) of Fig. 2. The full map of calculated densities can be found in the Supplementary Fig. S3. It is important to comment that the control over the coupling can be achieved only by the external laser field, keeping *w* fixed. In the rest of the article we will use *w* = 5 nm.

### 2-fold degeneracy induced by laser field and electric field effect

**Figure 3:**
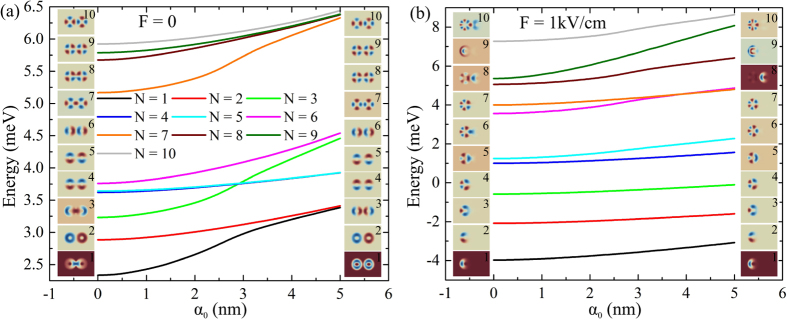
Energy spectrum dependence on *α*_0_ of the first ten states for two values of the electric field: *F* = 0 (**a**) and *F* = 1 Kv/cm (**b**). Inset columns relate to the forms of wave functions for the smallest and biggest values of *α*_0_.

As it is shown in Fig. 3, one can observe that the laser field also rearranges the energy spectrum. The dependence of the energy of the first ten dressed states on *α*_0_ and the wave functions for 0 and 5 nm values of *α*_0_ are presented in Fig. 3. The frames (a) and (b) are calculated for the absence and for a specific value of electric field strength, respectively. In both cases, one can observe that the energies increase when the field intensity increases too. This means that the energy levels are affected by the contraction of the dressed potential. In addition, the increment of *α*_0_ turns the energies of the ground and first excited states into a single 2-fold degenerated eigenvalue. These states can be considered as correspondingly bonding and antibonding ones. The new degeneracy is due to the decoupling that reforms bonding state to have the same probability density as the antibonding state, although the ground state has even parities P_*x*_ = 1 and P_*y*_ = 1 with respect to *x* and *y*-axis, while the excited one has P_*x*_ = −1 and P_*y*_ = 1. There are also four coupled pairs of bonding and antibonding states: 3rd and 6th, 4th and 5th, 7th and 10th, 8th and 9th. It is worth to mention that the 1st, 3rd, and 7th energy levels show non-linear variation, caused by the laser field induced decoupling, which, by localizing the states in the rings, increases the confinement. Meanwhile, the energies of the other states show almost linear alteration, since even in the absence of the laser field they are already localized in the rings. The degeneracy forced by the laser field vanishes if the electric field is applied, that ruins the reflection symmetry with respect to the *y*-axis and leaves only P_*y*_ = ±1 parity (see Fig. 3(b)). Electric field forces down almost all energy spectrum, as a consequence of tilted confining potential^52^. Only the highest 9th and 10th levels show increased values caused by the coupling with neighboring states.

### Intraband absorption in QR molecule affected by laser and electric fields

In order to qualitatively estimate the coupling of the states the additive absorption coefficient can be calculated. This coefficient for the transitions from the ground state to the excited states can be cast in the form^53^:

11 $$\alpha ({\rm{\Omega }})=\zeta \times \hslash {\rm{\Omega }}\sum _{f\mathrm{=2}}^{f\mathrm{=10}}{N}_{i,f}{|{M}_{i,f}|}^{2}\frac{{\rm{\Gamma }}}{{(\hslash {\rm{\Omega }}-{{\rm{\Delta }}}_{i,f})}^{2}+{{\rm{\Gamma }}}^{2}},$$

where Ω is the incident light angular frequency, ${N}_{i,f}={N}_{i}-{N}_{f}$ is the occupation difference of the initial and final states, ${M}_{i,f}$ denotes the dipole matrix element and ${{\rm{\Delta }}}_{i,f}={E}_{f}-{E}_{i}$. Here, ${N}_{i,f}\mathrm{=1}$ because the final state is always vacant and the initial one is occupied by one electron, the Lorentzian parameter Γ is taken as ${\rm{\Gamma }}=0.1\,{\rm{meV}}$, and *ζ* contains all the other factors^38^. Let us remark that the possibility to apply the expression (11) to study the intraband absorption is supported by the fact that in the high-frequency approximation the time-dependent problem (1) is reduced to an effective time-independent Schrödinger equation given by Eq. (9), which can be formally treated as the eigenvalue problem of a particle in a potential ${V}_{d}({{\bf{r}}}_{\perp },{\alpha }_{0})$ in the presence of electric field **F**. Similar formalisms have also been used in refs 38, 39, 54–56.

**Figure 4:**
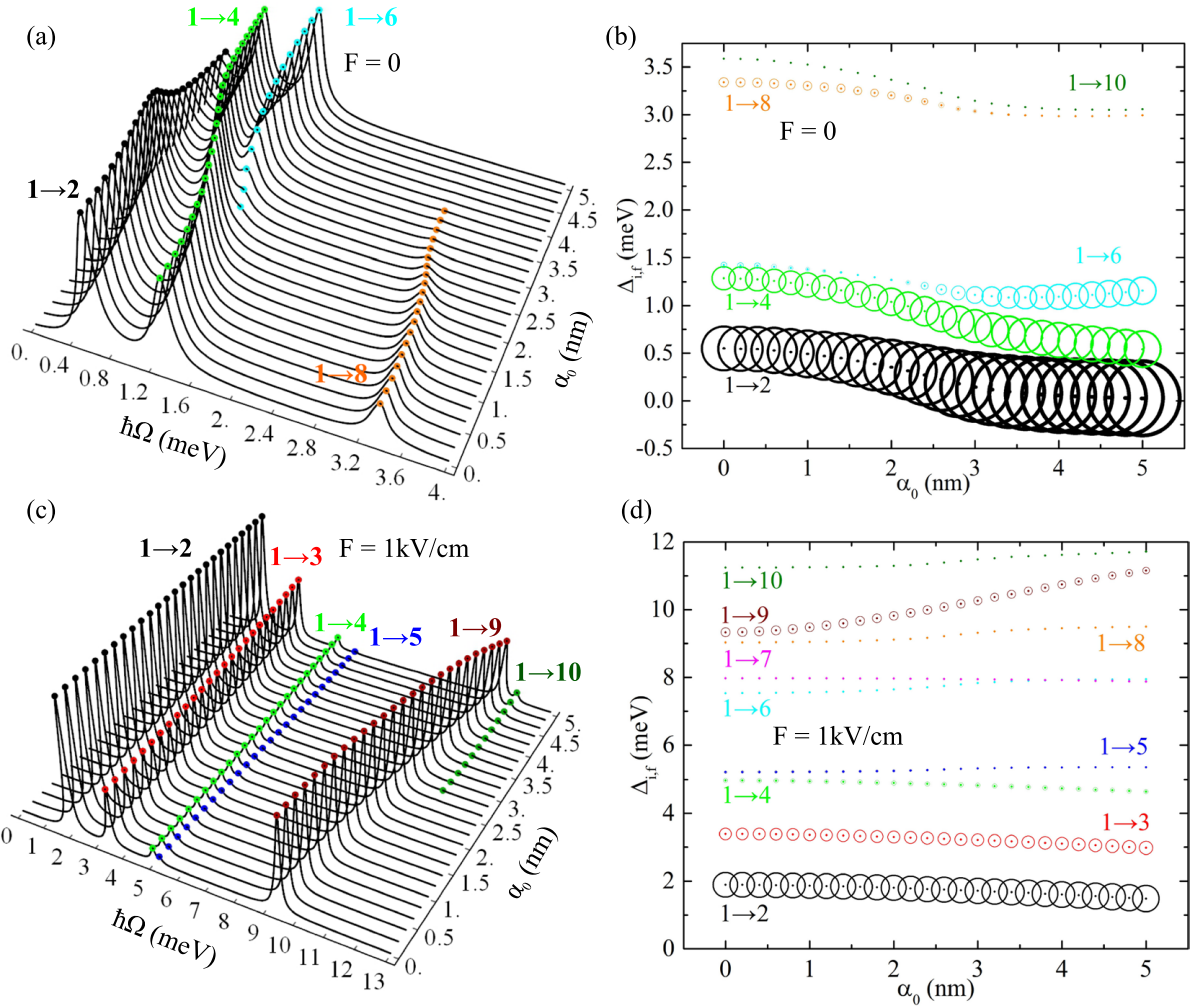
Absorption coefficient (in arbitrary units) dependence on incident photon energy *ħ*Ω for different values of *α*_0_ (Figs 3(c) and 4 (a)) and Δ_*i, f*_ energy difference dependence on *α*_0_ (Fig. 4(b) and (d)): the area of the circles is proportional to the respective transition probability ${|{M}_{i,f}|}^{2}$. The absence of electric field is considered in Fig. 4(a) and (b) and $F=1\,\mathrm{kV}/\mathrm{cm}$ in Fig. 4(c) and (d).

Absorption coefficient dependence on the incident photon energy is presented in Fig. 4(a) and (c), considering circularly polarized light falling perpendicularly to the plane of the rings. Also, the ${{\rm{\Delta }}}_{i,f}$ dependence on *α*_0_ is presented in Fig. 4(b) and (d), where the area of the circles is proportional to the corresponding transition probability ${|{M}_{i,f}|}^{2}$. As presented in Fig. 4(a), when $F=0$ the transitions are allowed only to the 2nd, 4th, 6th, 8th and 10th states with non-equal parities: for the 2nd, 6th and 10th ${P}_{x}=-1$ and ${P}_{y}=1$ for the 4th and 8th *P*_*x*_ = −1 and *P*_*y*_ = −1 (see the wave functions in Fig. 3(a)). The other states have *P*_*x*_ = −*P*_*y*_, which means that the selection rule is dependent on the symmetry of the wave functions. From all the allowed transitions the biggest probability has 1 → 2, although it is not the only decisive factor to estimate the value of the absorption coefficient. The probability of 1 → 4 transition is obviously smaller than of 1 → 2 one, but nevertheless, for bigger *α*_0_ the former has bigger absorption, that is a consequence of the Ω factor in Eq. (11). A similar phenomenon is observed for the *α*_0_ values close to 5 nm: 1 → 4 with greater probability leads to the lesser absorption compared with 1 → 6. In addition, the absorption spectrum is both red- and blueshifted only for 1 → 6 transition; in other cases the redshift is observed. 1 → 10 transition, although allowed, has very small probability, and becomes invisible in the absorption spectrum. The presence of the electric field (1 kV/cm) eliminates the symmetry with respect to the *y*-axis, allowing all the transitions. In fact, in Fig. 4(d), the greatest probability has 1 → 2 transition, although the transition to farther-positioned 9th level demonstrates absorption of the same order. Now, absorption spectrum in Fig. 4(c) redshifts for the transitions to the 2nd, 3rd, 4th states, and blueshifted with 1 → 5, 1 → 9 and 1 → 10 transitions. Those to 6th, 7th, and 8th have a much less contribution to the absorption spectrum.

### Electric field direction effect on molecular spectrum

**Figure 5:**
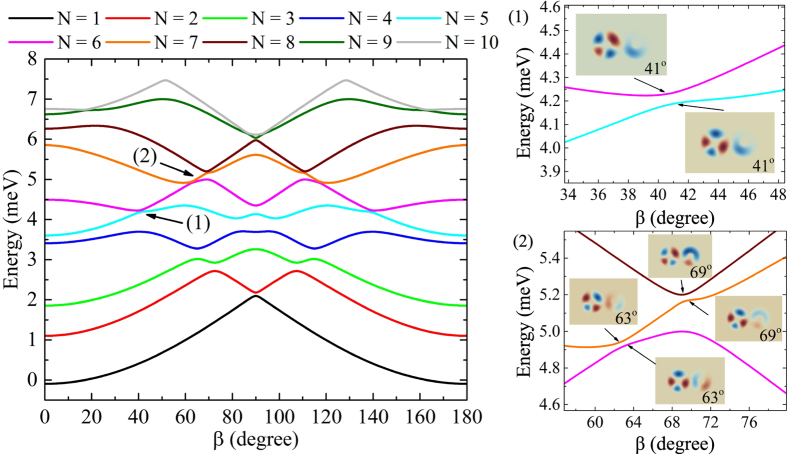
Energy spectrum dependence on the electric field direction at $F=0.5\,\text{kV}/\text{cm}$ and ${\alpha }_{0}=2.5\,{\rm{nm}}$. Figure 5(1) and (2) show energy variation around the anti-crossing-like points.

Given the symmetry effects on QR molecule caused by laser and electric fields applied along the fixed directions, the variation of the angle $\beta$ between these two fields is considered. In particular, this angle is defined as $\beta =\angle (\widehat{{\bf{u}}},{\widehat{{\bf{e}}}}_{x})$, where is ${\widehat{{\bf{e}}}}_{x}$ is laser axis and $\widehat{{\bf{u}}}$ is the unit vector of electric field. We assume that $F=0.5\,\text{kV}/\text{cm}$ and ${\alpha }_{0}=2.5{\rm{nm}}$. As we can observe in Fig. 5, the variation of *β* creates different distributions of wave functions that cause oscillations of the energy spectrum. Notice that the energy spectrum is observed to be symmetric with respect to *β* = 90° value. This phenomenon is a consequence of the symmetry of the dressed potential ${V}_{d}({{\bf{r}}}_{\perp },{\alpha }_{0})$ with respect to *x*- and *y*- axis. This issue can be observed with the help of the Supplementary Fig. S2, where the effect of electric field direction on the dressed potential is presented.

On interesting feature of the angular dependence is the emergence of *anti-crossing-like points*. In general, one can refer to an anti-crossing point as a point where the energies levels are very close, such that the corresponding wave functions have the same symmetry^57^. Hence, we refer to an anti-crossing-like point as a point where the energies levels are very close and the corresponding wave functions have similar shape. Some of the anti-crossing-like points are shown in Fig. 5(1) and (2). For instance, one can observe in Fig. 5(2) that at the angles 63° and 69° the wave functions have similar shape. The occurrence of these anti-crossing-like behaviors is rather unexpected, since they happen when an electric field is not applied along any coordinate axis. We remark that a similar study has been performed by Baimuratov *et al*.^58^, where the impurity levels showed anti-crossings with the increment of the nanocrystal radius. The authors have shown that the property is inherent to the energy spectra of charge carriers whose spatial motion is simultaneously affected by the Coulomb potential of the impurity ion and the confining potential of the nanocrystal, while we achieve anti-crossing-like behaviors by rotating the electric field and keeping the laser field polarization ${\widehat{{\bf{e}}}}_{x}$ and parameter *α*_0_ fixed. The wave functions at all the anti-crossing-like points and for other value of the electric field amplitude are included in Supplementary Figs S4 and S5, respectively.

### Intraband absorption affected by electric field direction

**Figure 6:**
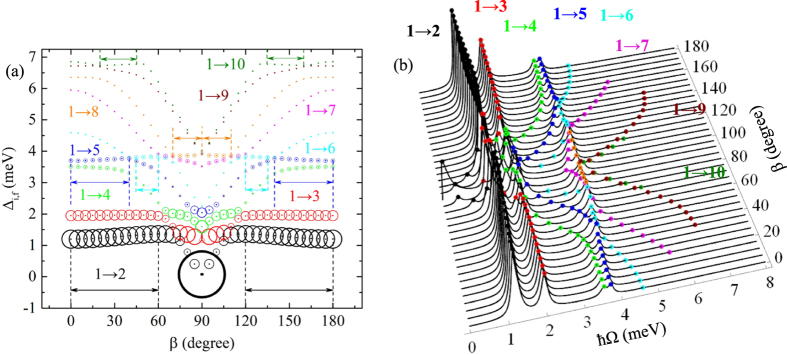
Δ_*i, f*_ energy difference dependence on *β* angle (Fig. 6(a)) and absorption coefficient (in arbitrary units) dependence on incident photon energy *ħ*Ω for different directions of $F=0.5\,\text{kV}/\text{cm}$ electric field defined by *β* and for *α*_0_ = 2.5 nm (Fig. 6(b)). The area of the circles in Fig. 6(a) is proportional to the respective transition probability ${|{M}_{i,f}|}^{2}$.

Considering different values of *β* the energy difference, ${{\rm{\Delta }}}_{i,f}$, and the corresponding absorption spectrum are illustrated in the frame (a) and (b) of Fig. 6, respectively. Since the energy levels on *β* have mirror symmetry with respect to *β* = 90, implies that ${{\rm{\Delta }}}_{i,f}$ energy differences are also symmetric as we can observe in Fig. 6(a). This feature turns to make a symmetric absorption spectrum as it is shown in Fig. 6(b). The presence of electric field allows all the transitions from the ground state like in Fig. 4(d). The absorption spectrum redshifts in $[\mathrm{0,90}]$ interval and blueshifts in [90°, 180°] with the following exceptions (shown in Fig. 6(a)): blue(red)shift in [0°, 60°]([120°, 180°]) for 1 → 2, [0°, 40°]([140°, 180°]) for 1 → 5, [45°, 60°]([120°, 135°]) for 1 → 6, [70°, 90°]([90°, 110°]) for 1 → 8 and [20°, 45°]([135°, 160°]) for 1 → 10. In comparison with Fig. 4(a) and (b), here the absorption peaks are distributed in a much more complex way, resulting from the oscillating ${{\rm{\Delta }}}_{i,f}$ and the anti-crossing-like points. Nevertheless, one can observe that the biggest contributions are from 1 → 2, 1 → 3, and 1 → 4 transitions.

## Final Remarks

In conclusion, we have shown that the artificial molecule, modeled by two lateral QRs, can be controlled by an intense laser field, up to its total decoupling and the energy spectrum of the molecule obtains 2-fold degeneracy. We suggest the decoupling QRs only with the external laser influence, keeping the overlapping (material) size of the QRs unchanged. It is alternative to the decoupling by the material separation of QRs, proposed in ref. 18. We would like to point out there is an interesting connection between the degeneracy obtained in our work with the collapse of minibands in a superlattice irradiated with the intense THz radiation^59^. The collapse occurs if the ratio of the Bloch frequency $-e{E}_{0}d$ (where *e* is the electron charge, *E*_0_ is the strength of the laser field and *d* is the superlattice period) and the laser field angular frequency *ω* becomes equal to zero of the Bessel function *J*_0_. In such situation, an increment of the value of *E*_0_ results in the vanishing of the collapse effect and therefore the energies become non-degenerated. While in our molecular system of QRs, we have achieved and kept the levels degenerated when the value of laser field strength is increased. This phenomenon is produced because the amplitude of the quiver motion vector *α*_0_ is directly proportional to *E*_0_. Furthermore, in ref. 59 the collapse is attributed to the spatial periodicity of the superlattice and the temporal periodicity of the external laser field, whereas in our manuscript we have demonstrated that the degeneracy induced by intense laser field can be achieved in a molecular system (composed of laterally coupled pair of quantum rings), which is not periodic in space.

We have also shown that the lateral electric field interferes significantly with laser field induced degeneracy. In fact, the electric field causes the elimination of the degeneracy as well as opens new channels for optical transitions. The angular variation of electric field creates unexpected anti-crossing-like behavior points that are responsible for the oscillations in the energy spectrum. Furthermore, both blue and redshifts of the intraband absorption spectrum have been obtained, which are influenced by laser and electric fields.

Finally, let us comment that recently has been published an article concerning the experimental observations of the delocalization - localization phenomenon in a GaAs quantum Hall system induces by light^60^. This interesting result can motivate for a deep examination of the whole spectrum of the bound states of quantum ring superlattices under the influence of THz radiation and magnetic fields. This will be our next step of studies.

## Methods

The equation (9) is numerically solved using finite element analysis with the partial differential module of COMSOL Multiphysics^61^. Triangular meshing has been chosen, with the 4^*th*^ order Lagrangian shape functions^62^. The dimensions of the meshing regions are taken large enough to avoid the leaking out of all the considered eigenstates. In addition, as long as the direction of the electric field is not kept fixed in the work, the mesh follows the symmetry established by the laser field. Namely, the whole meshing region is divided into four sections; once one section is meshed, its meshing is copied into the adjacent sections, keeping the symmetry with respect to the *x*- and *y*- axis. An example of meshing style, along with the values of meshing parameters is presented in the Supplementary Fig. S6.

## Electronic supplementary material

Supplementary Information
