# Supplementary material for: Molecular spectrum of laterally coupled quantum rings under intense terahertz radiation
Source: Sci Rep. 2017 Sep 5;7:10485. doi: 10.1038/s41598-017-10877-y (PMC5585341; doi:10.1038/s41598-017-10877-y)
Supplement: Supplementary file 1 — Supplementary Information [file 41598_2017_10877_MOESM1_ESM.pdf]

Supplementary Information for "Molecular spectrum of laterally coupled quantum rings under intense terahertz radiation"

Henrikh M. Baghramyan[1,*], Manuk G. Barseghyan[2] and David Laroze[1,3]

[1]Instituto de Alta Investigación, CEDENNA, Universidad de Tarapacá, Casilla 7D, Arica, Chile.

[2]Department of Solid State Physics, Yerevan State University, Alex Manoogian 1, 0025 Yerevan, Armenia.

[3]Yachay Tech University, School of Physical Sciences and Nanotechnology, 00119-Urcuquí, Ecuador.

[*]hbaghramyan@uta.cl

In this supplementary information we have included the following figures. In Fig. S1 we present cross-sectional view of the $V_d^F(\mathbf{r}_\perp, \alpha_0, \mathbf{F}) = V_d(\mathbf{r}_\perp, \alpha_0) - e\mathbf{F}\cdot\mathbf{r}_\perp$ potential considering different values of electric field strength F and fixed values of laser field parameter α_0 and angle β that defines the direction of electric field. Meanwhile, in Fig. S2 three-dimensional graph of $V_d^F(\mathbf{r}_\perp, \alpha_0, \mathbf{F})$ is shown for various β and fixed α_0 and F.

Fig. S3 depicts electron ground state's probability density considering different quantum ring overlapping distance w and laser field parameter α_0.

Figs. S4 and S5 are demonstrated to show all the anti-crossing-like points that appear in the energy spectrum of an electron influenced by changing the direction of the electric field $F = 0.5$kV/cm and $F = 1$kV/cm, correspondingly. Laser field polarization direction is kept fixed along the x-axis and $\alpha_0 = 2.5$nm.

Fig. S6 shows an example of mesh that has been used for the numerical calculations.

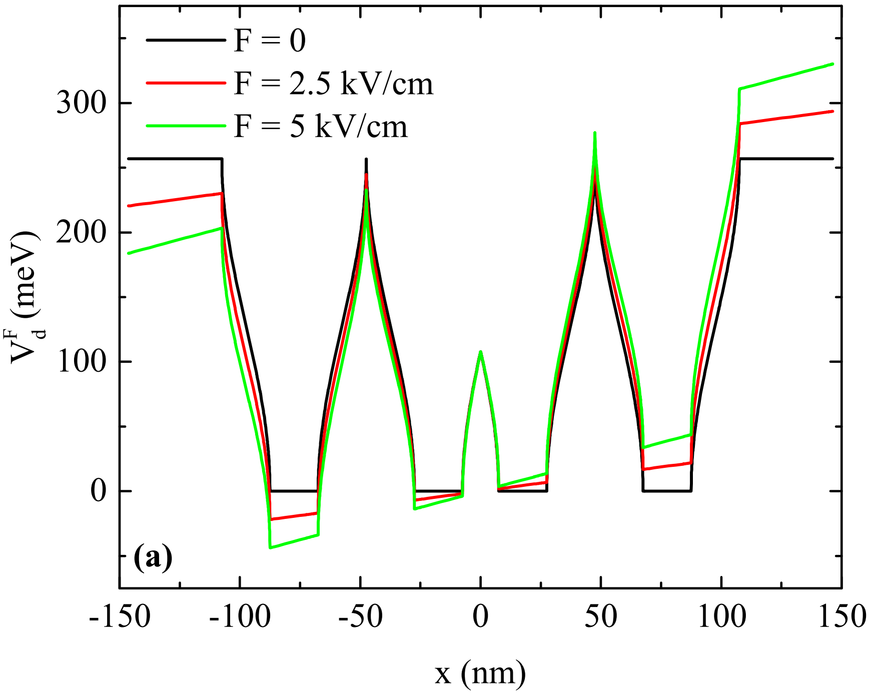

Figure S1: Cross-sectional view of the $V_d^F(\mathbf{r}_\perp, \alpha_0, \mathbf{F})$ potential for different values of F at $\alpha_0 = 10$nm and $\beta = 0°$

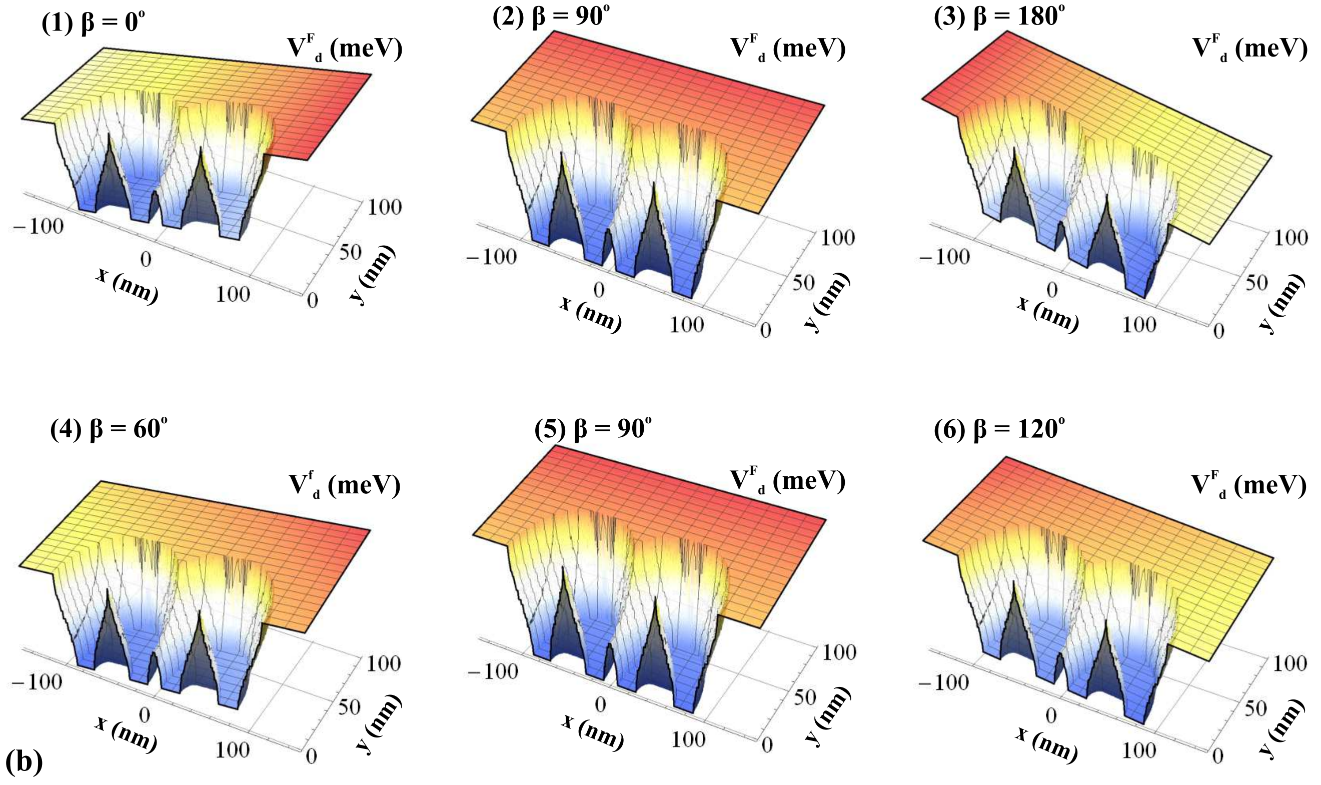

Figure S2: Three-dimensional view of the $V_d^F(\mathbf{r}_\perp, \alpha_0, \mathbf{F})$ potential for different values of β at $\alpha_0 =$ 10nm and $F = 5$kV/cm

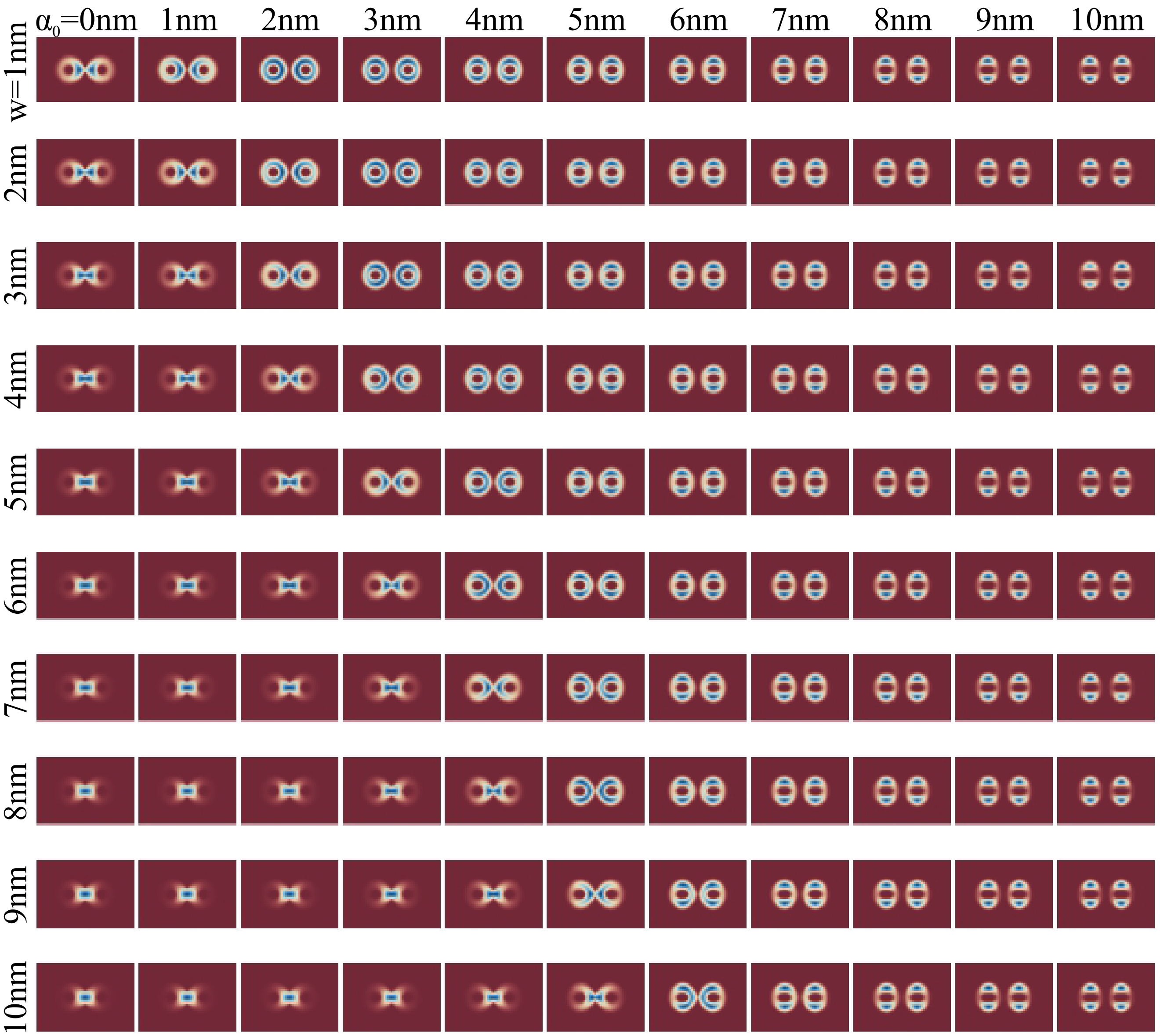

Figure S3: The full map of probability density of the ground state for different values of w and α_0.

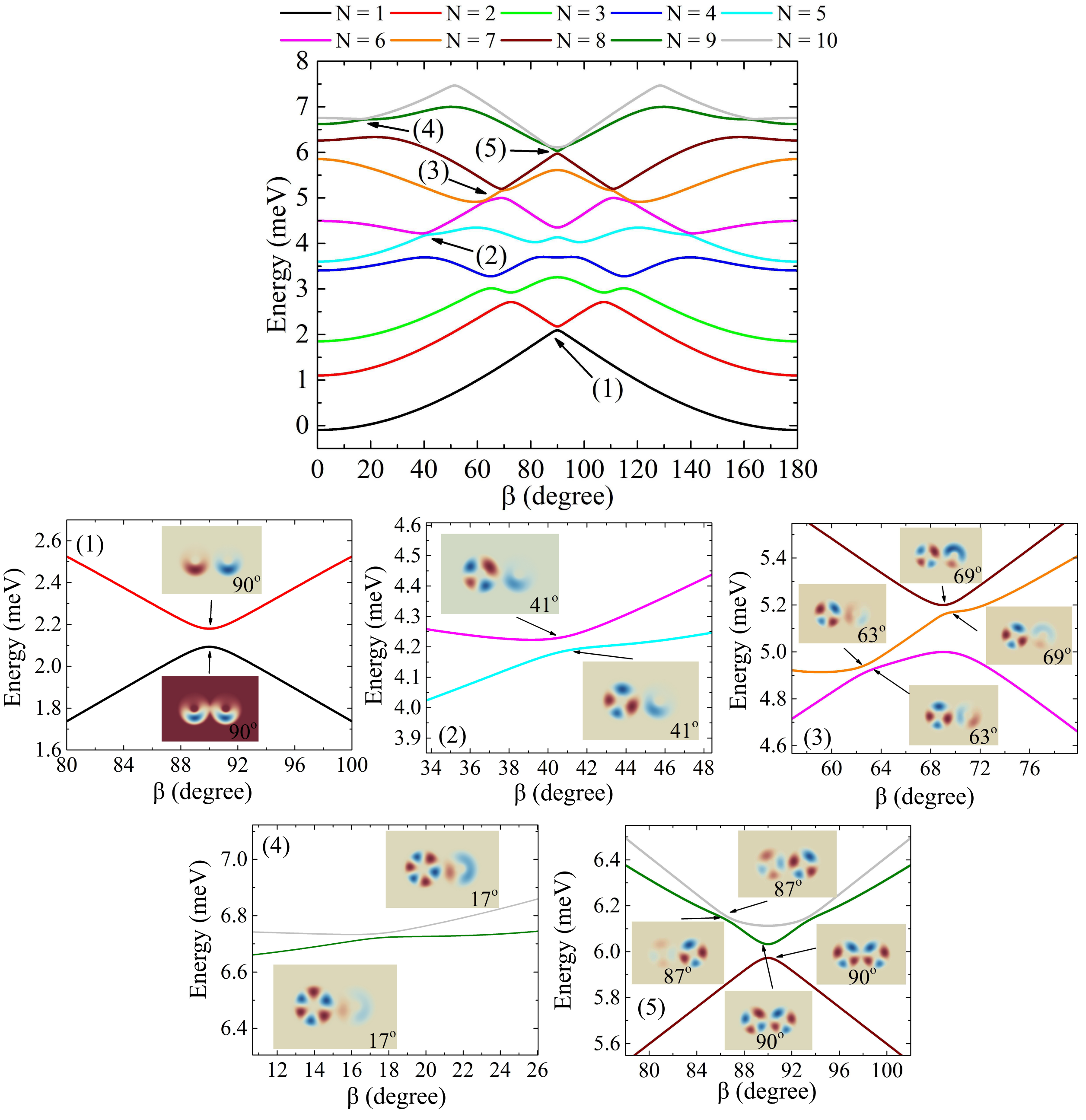

Figure S4: Energy spectrum dependence on $F = 0.5$kV/cm electric field direction for $\alpha_0 = 2.5$nm. Figs. (1)-(5) show energy variation around the anti-crossing-like points.

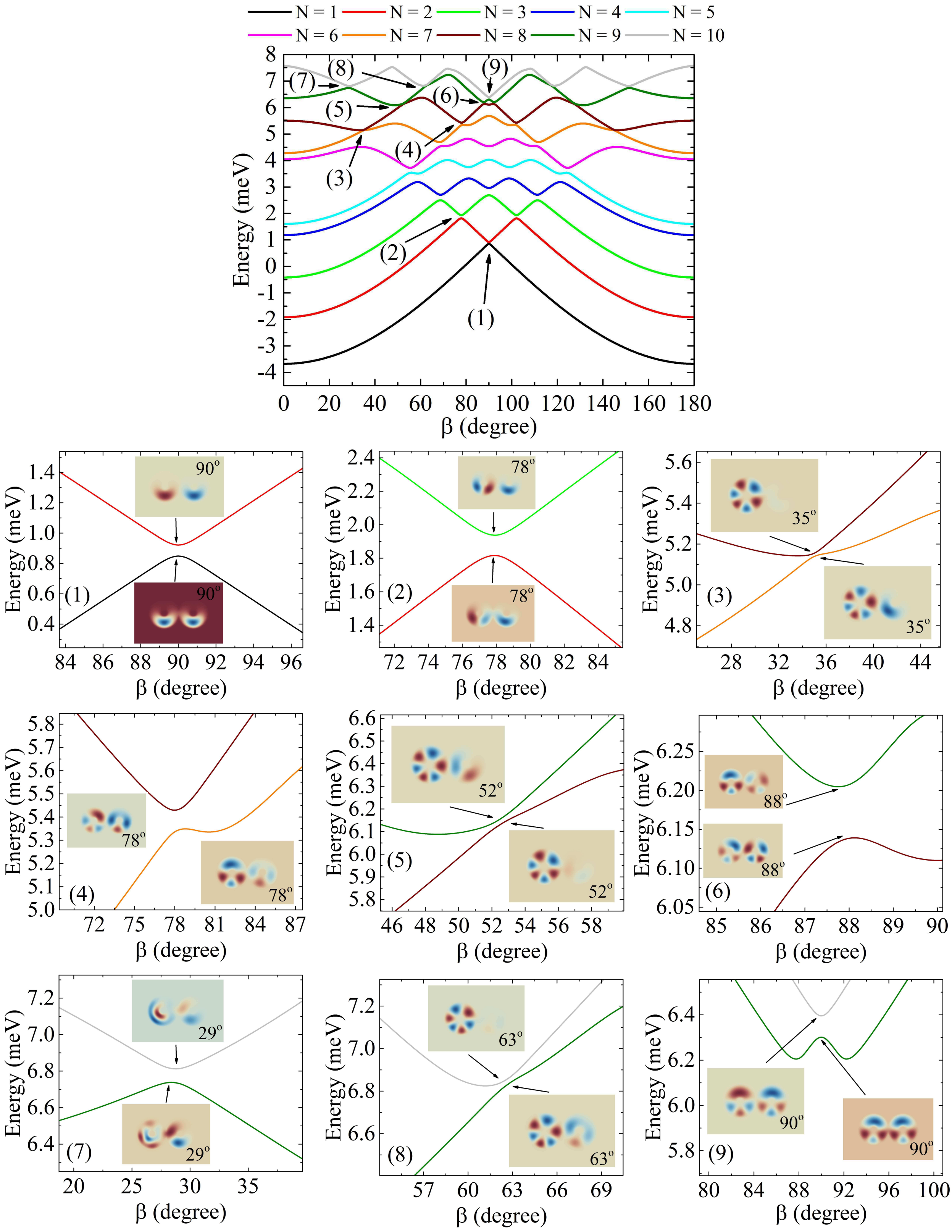

Figure S5: Energy spectrum dependence on $F = 1\text{kV/cm}$ electric field direction for $\alpha_0 = 2.5\text{nm}$. Figs. (1)-(9) show energy variation around the anti-crossing-like points.

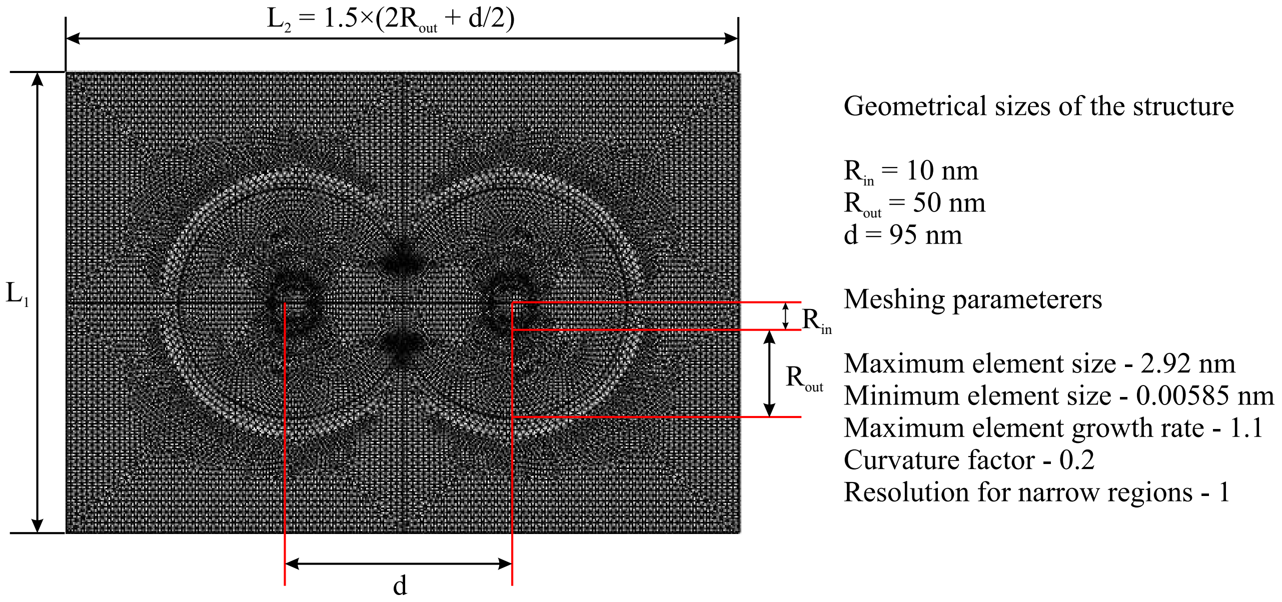

Figure S6: An example of the meshing, done for the numerical calculations.
